# The Narrow Escape From Ice Pick Penetration Sparing Neck’s Vital Structures: A Case Report

**DOI:** 10.7759/cureus.88046

**Published:** 2025-07-15

**Authors:** Pratik Kumar, Prince Handa, Ashish Gopal, Aayushi Parasar, Anubhuti Gupta

**Affiliations:** 1 Otolaryngology - Head and Neck Surgery, Maulana Azad Medical College, New Delhi, IND; 2 Otolaryngology, Maulana Azad Medical College, New Delhi, IND

**Keywords:** cervical foreign body, ice pick injury, neck exploration, penetrating neck trauma, sharp metallic foreign body

## Abstract

Penetrating neck injuries carry a high risk of morbidity and mortality due to the concentration of vital structures within a confined space. We present a rare case of a 21-year-old woman who sustained a homicidal assault with a metallic ice pick that penetrated obliquely through the neck, passing perilously close to the carotid sheath, trachea, esophagus, and major nerves, yet without damaging any vital structures. Surgical exploration and removal were successfully performed under general anesthesia. Postoperatively, the patient developed left vocal cord paresis, which was managed conservatively. This case underscores the importance of prompt imaging, meticulous surgical planning, and a multidisciplinary approach in managing complex penetrating neck injuries.

## Introduction

Penetrating neck trauma constitutes approximately 10% of all traumatic injuries and presents significant challenges due to the compact anatomical region containing critical structures such as the trachea, esophagus, carotid and jugular vessels, thyroid gland, spinal cord, and multiple cranial and peripheral nerves [1]. Even seemingly minor injuries can be life-threatening. While knife injuries are well documented, impalements with unusual objects like ice picks are exceedingly rare. Their slender, pointed nature may deceptively spare vital structures, creating a high-risk scenario with limited external signs. This case report details oblique transcervical impalement by an ice pick that traversed dangerously close to vital structures, yet caused no major vascular or aerodigestive damage. The case highlights the life-saving importance of timely imaging, airway protection, and structured surgical exploration.

## Case presentation

**Figure 1:**
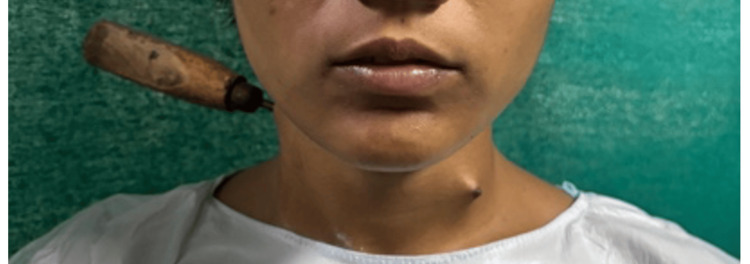
Clinical image depicting oblique course of ice pick

A 21-year-old woman presented to the emergency department following a homicidal assault with a metallic ice pick. She reported severe neck pain but denied bleeding, respiratory distress, hoarseness, or neurological deficits. On examination, she was conscious, hemodynamically stable, and breathing comfortably. A metallic foreign body was visibly lodged on the right side of her neck at the level of the hyoid bone, with the tip tenting the skin on the left side near the first tracheal ring, suggesting an oblique trajectory (Figure 1).

**Figure 2:**
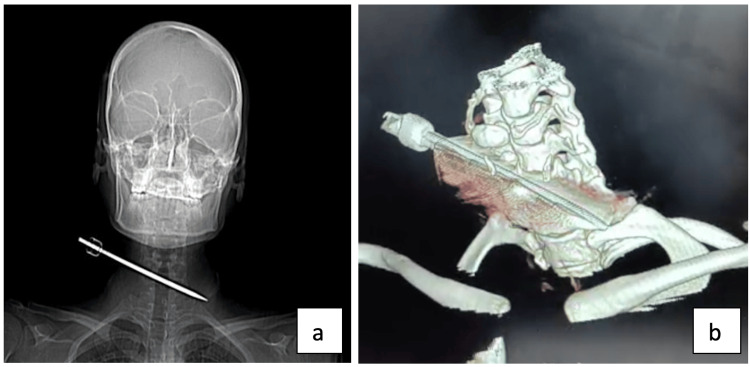
CECT neck depicting the foreign body: (a) coronal view and (b) 3D reconstruction CECT: Contrast-enhanced computed tomography.

Contrast-enhanced computed tomography (CT) of the neck revealed the ice pick coursing posterior to the right carotid sheath, trachea, and esophagus, crossing the left tracheoesophageal groove, and piercing the left thyroid lobe without injuring nearby vessels or the airway, with coronal view (Figure 2, Panel a) and 3D reconstruction (Figure 2, Panel b) confirming its perilous path.

The patient underwent general anesthesia with video laryngoscope-guided endotracheal intubation. Fiber-optic laryngoscopy and tracheoscopy showed no airway injury; esophagoscopy confirmed an intact hypopharynx and esophagus.

**Figure 3:**
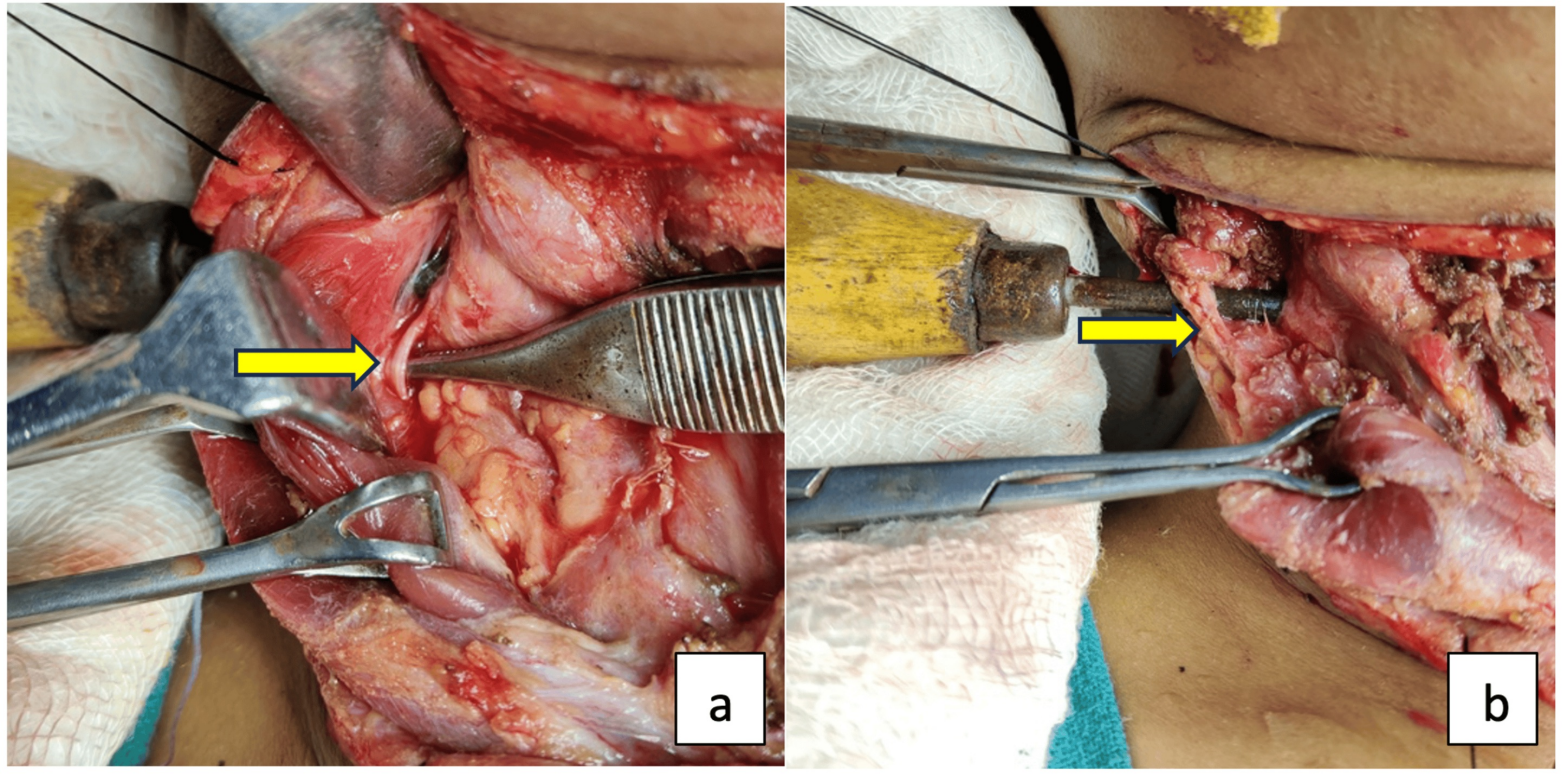
Intraoperative image showing (a) the spinal accessory nerve and (b) the greater auricular nerve

A transverse cervical incision incorporating entry and exit sites was made. Subplatysmal flaps were raised, and bilateral sternocleidomastoid and strap muscles were dissected, preserving the spinal accessory nerve (Figure 3, Panel a) and greater auricular nerve (Figure 3, Panel b) on the right side.

**Figure 4:**
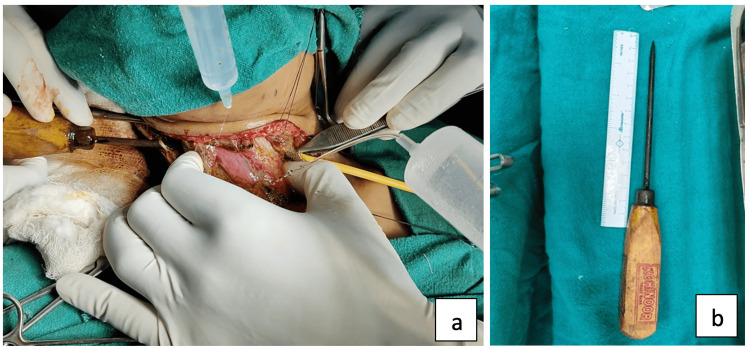
(a) Intraoperative removal of icepick with its tip covered by a Foley's catheter and (b) extracted ice pick

The ice pick was gently extracted from the right side using a controlled twisting technique after lubricating with liquid paraffin, lignocaine jelly, and Amikacin solution. The tip was capped with a Foley’s catheter to prevent iatrogenic injury during removal (Figure 4, Panel a), and the foreign body was delivered uneventfully (Figure 4, Panel b).

Hemostasis was ensured with the Valsalva maneuver. A neck drain was placed, and the wound was closed in layers. Post-extubation, the patient developed hoarseness. Indirect laryngoscopy showed left vocal cord paresis with preserved right cord mobility. Speech therapy and steroids were initiated, and complete recovery was achieved on day 10. Psychiatric evaluation diagnosed adjustment disorder, for which escitalopram and clonazepam were prescribed. The patient showed gradual improvement and was discharged with normal swallowing and recovering phonation.

## Discussion

Penetrating neck injuries are fraught with danger due to the density of vital structures in a small area. Life-threatening injuries to the carotid and jugular vessels, trachea, esophagus, or cranial nerves can lead to rapid deterioration [2]. The anatomical division of the neck into zones helps guide evaluation and intervention. Zone II (between the cricoid cartilage and angle of the mandible), where this injury occurred, is the most commonly affected and the most accessible for surgical exploration [1].

While vascular injuries occur in up to 25% of cases [1] and aerodigestive injuries in 30% [1,3], our patient miraculously avoided such complications. This case underscores the unreliability of physical examination alone; although the patient was stable and asymptomatic externally, contrast-enhanced CT was essential for determining the trajectory and assessing structural involvement.

CT angiography is now a cornerstone in trauma imaging, providing high-resolution detail that aids surgical planning [4]. Airway protection via video laryngoscopy was crucial to minimize further trauma during anesthesia induction. The decision to surgically explore and remove the object under direct visualization aligns with trauma protocols, as blind removal could result in catastrophic hemorrhage or airway collapse [5].

Intraoperative visualization allowed controlled dissection and foreign body retrieval without injury. The postoperative left vocal cord paresis likely resulted from transient neuropraxia of the recurrent laryngeal nerve. Conservative management with speech therapy led to gradual recovery, illustrating the importance of follow-up in such injuries.

This case highlights the critical role of imaging, structured exploration, and a coordinated multidisciplinary approach in managing rare and potentially fatal penetrating neck injuries.

## Conclusions

This case demonstrates how deeply penetrating neck injuries may, by sheer trajectory, avoid damaging vital structures. Successful management hinges on prompt imaging, careful airway control, avoidance of blind extraction, and meticulous surgical dissection. A thorough understanding of cervical anatomy and adherence to trauma protocols are essential for optimizing patient outcomes in complex neck trauma.
